# Common Genetic Variants Are Associated with Accelerated Bone Mineral Density Loss after Hematopoietic Cell Transplantation

**DOI:** 10.1371/journal.pone.0025940

**Published:** 2011-10-14

**Authors:** Song Yao, Lara E. Sucheston, Shannon L. Smiley, Warren Davis, Jeffrey M. Conroy, Norma J. Nowak, Christine B. Ambrosone, Philip L. McCarthy, Theresa Hahn

**Affiliations:** 1 Department of Cancer Prevention and Control, Roswell Park Cancer Institute, Buffalo, New York, United States of America; 2 Department of Medicine, Roswell Park Cancer Institute, Buffalo, New York, United States of America; 3 Department of Cancer Genetics, Roswell Park Cancer Institute, Buffalo, New York, United States of America; 4 Department of Biochemistry, University at Buffalo, Buffalo, New York, United States of America; Harvard University, United States of America

## Abstract

**Background:**

Bone mineral density (BMD) loss commonly occurs after hematopoietic cell transplantation (HCT). Hypothesizing that genetic variants may influence post-HCT BMD loss, we conducted a prospective study to examine the associations of single nucleotide polymorphisms (SNP) in bone metabolism pathways and acute BMD loss after HCT.

**Methods and Findings:**

We genotyped 122 SNPs in 45 genes in bone metabolism pathways among 121 autologous and allogeneic HCT patients. BMD changes from pre-HCT to day +100 post-HCT were analyzed in relation to these SNPs in linear regression models. After controlling for clinical risk factors, we identified 16 SNPs associated with spinal or femoral BMD loss following HCT, three of which have been previously implicated in genome-wide association studies of bone phenotypes, including rs2075555 in *COL1A1*, rs9594738 in *RANKL*, and rs4870044 in *ESR1*. When multiple SNPs were considered simultaneously, they explained 5–35% of the variance in post-HCT BMD loss. There was a significant trend between the number of risk alleles and the magnitude of BMD loss, with patients carrying the most risk alleles having the greatest loss.

**Conclusion:**

Our data provide the first evidence that common genetic variants play an important role in BMD loss among HCT patients similar to age-related BMD loss in the general population. This infers that the mechanism for post-HCT bone loss is a normal aging process that is accelerated during HCT. A limitation of our study comes from its small patient population; hence future larger studies are warranted to validate our findings.

## Introduction

After the second decade of life, bone mineral density (BMD) gradually declines as a result of normal aging [1]. This process is accelerated in patients undergoing hematopoietic cell transplantation (HCT) [2], [3]. Rapid bone loss weakens bone strength and may permanently impair bone remodeling capability [4], [5], putting HCT survivors at high risk of premature fracture. We previously demonstrated that BMD loss within 4 months after HCT is equivalent to 7 to 17 years of normal bone aging [6]. Moreover, this loss occurs following autologous and allogeneic HCT with a similar incidence and severity.

In the general population, the inherited genetic background accounts for a large proportion of phenotypic variation in BMD loss and fracture risk [7], [8]. In numerous candidate gene association studies, a variety of genetic variants, mostly in the form of single nucleotide polymorphisms (SNP), have been associated with regulation of BMD and risk of fracture [9], with less than a handful of them confirmed in a recent large-scale meta-analysis performed by the Genetic Factors for Osteoporosis (GEFOS) Consortium [10]. A genome-wide association (GWA) approach has also been used to search for loci associated with various bone phenotypes, which identified a number of novel SNPs [11], [12], [13], [14], [15]. These genetic markers may have clinical significance in predicting risk of BMD loss and/or osteoporosis. Moreover, some may also be predictive for response to therapies targeting related molecules or pathways, such as a human monoclonal antibody against receptor activator of nuclear factor κB ligand (RANKL) (denosumab), which was recently approved by the FDA for postmenopausal women at risk for fracture [16].

Among HCT recipients, rapid BMD loss may be due to an accelerated bone aging process after exposure to high doses of corticosteroids, chemotherapy and/or total body irradiation. Indeed, we have previously demonstrated that bone loss occurs within three months of HCT in both allogeneic and autologous HCT patients [6]. Therefore, predisposing genetic variants for low BMD and osteoporosis in the general population may also predispose carriers to risk of severe post-HCT BMD loss. Recently, McClune and colleagues proposed comprehensive guidelines for the screening, prevention and treatment of osteoporosis after HCT. They recommend dual-energy X-ray absorptiometry (DXA) scans for all adult patients 1 year post-HCT or earlier for patients at high risk of bone loss [17]. Bisphosphonates are effective in reducing or reversing HCT-related bone loss in several small randomized studies; however, they rarely resolve bone loss to pre-HCT levels and have a significant side-effect, osteonecrosis of the jaw. We hypothesized that genetic variants are associated with BMD loss in HCT patients, and sought to identify a panel of genetic biomarkers that can be incorporated into the above guidelines to personalize prevention and treatment strategy.

## Methods

*Ethics statement:* Stem cell transplant patients at the Blood and Marrow Transplantation Program included in this study are part of an institution-level Data Bank and Biorepository (DBBR) at Roswell Park Cancer Institute (RPCI). All participants have provided written consent for their data and biospecimens to be used for research purposes. This study has been reviewed and approved by RPCI Institutional Review Board.

### Patients, BMD measurement, and DNA specimens

A detailed description of the patient population has been published previously [6]. Briefly, between 1/2006 and 1/2009, 206 consecutive adult (≥18 years) patients underwent their first HCT in the Blood and Marrow Transplantation Program at Roswell Park Cancer Institute (RPCI). As part of routine clinical care, the average BMD at lumbar spine (L2–L4) and dual femurs were measured by DXA scans at baseline (pre-HCT) and day +100 post-HCT. The median time for 197 patients (96%) who had a baseline DXA scan was 20 days before HCT, and 98 days for 146 patients (74%) who had a second DXA scan after HCT. Reasons for not obtaining a follow-up DXA scan were unstable medical status (n = 14), or disease relapse/early death (n = 37). Before HCT, patients were invited to participate in the DataBank and BioRepository (DBBR), a comprehensive data and biospecimen core facility at RPCI [18]. Upon their consent to the DBBR, blood samples were drawn and genomic DNA was extracted and stored for multidisplinary research. For this study, 121 patients including 67 autologous and 54 allogeneic HCT patients, consented to the DBBR and had adequate DNA available for genotyping.

### Candidate gene and SNP selection and genotyping

Candidate genes from bone metabolism pathways were selected based on the following criteria: (1) genes that have been well studied and established in association with osteoporosis or other bone phenotypes in the general population; (2) key genes in bone metabolism pathways, including osteoblast or osteoclast differentiation, proliferation and activation; calcitronic hormone metabolism and activation; and bone matrix formation and regulation. As listed in Table S1, a total of 45 candidate genes were clustered into 4 groups based on underlying biological function, including (1) RANKL-RANK-OPG central signaling axis and regulating cytokines, (2) bone matrix proteins and regulating factors, (3) vitamin D receptor and metabolizing enzymes, and (4) steroid hormones and receptors.

SNPs for each candidate gene were selected based on the following criteria: (1) minor allele frequency (MAF) ≥0.10 in the population of European ancestry; (2) SNPs that have been well studied and established in association with osteoporosis or other bone traits in the general population, including those identified in recent GWA studies and the large prospective study Genetic Factors for Osteoporosis (GEFOS) Consortium [10]; (3) coding SNPs and SNPs in the 3′ or 5′ untranslated region (UTR); and (4) tagSNPs selected by the Tagger program [19] from the HapMap Project data [20], if no functional SNPs or well studied SNPs were available. In all, 158 SNPs were initially selected from the 45 candidate genes. To remove redundant SNPs due to high linkage disequilibrium (LD), pair-wise *r^2^* was computed for SNPs in each gene and in genes clustered on the same chromosome by PLINK [21]; SNP_tools [22] was used for necessary data formatting. For SNP pairs in high LD (*r^2^*≥0.90), one SNP was randomly selected from each pair for further consideration. As a result, a total of 122 SNPs were included in the final analysis (see Table S1).

Genotyping of DNA extracted from peripheral blood was performed in the Genomics Shared Resource at RPCI utilizing the MassARRAY® technology and iPLEX Gold assay (Sequenom). Nine percent duplicate samples and in-house Coriell trio samples were included within plates and across plates for genotyping quality assurance. The average qualified call rate was 98.1% for each SNP and 98.3% for each DNA sample. There was no discordance among duplicate pairs, and no SNPs violated Mendelian inheritance or Hardy-Weinberg equilibrium.

### Statistical analysis

All analyses were performed stratified by autologous and allogeneic HCTs since these two treatments, and the characteristics of the patients given these two treatments, are significantly different. Change in BMD was standardized to 100 days (BMD change divided by the number of days between the two DXA scans then times 100) and used as a continuous variable; thus, a negative value indicates BMD loss and a positive value indicates BMD gain. We used linear regression models to analyze the association of clinical factors and genetic variants with four separate outcomes: BMD changes at the spine and at the femur in autologous and allogeneic HCT patients. Assuming an additive genetic model, 54 patients in the small allogeneic HCT group and two-sided p-value of 0.05, we had 80% power to detect a minimum BMD change of 0.023–0.040 g/cm^2^ for a SNP with a minor allele frequency between 0.10–0.50. In the autologous HCT group which had a larger sample size of 67, we had 80% power to detect effect sizes <0.023 g/cm^2^.

#### Single SNP analysis

SNPs associated with HCT-induced BMD change were identified as follows. First, each SNP was tested individually in a simple linear regression model without controlling for covariates; SNPs with p<0.10 were then added to regression models previously built with clinical factors for each of the four outcomes, including the cumulative dose of corticosteroids (in prednisone equivalents) between the two DXA scans for BMD loss in allogeneic HCT patients [6]. We measured the proportion of variance explained by all the covariates retained in a model, *R^2^*, and determined, *ΔR^2^*, an estimate of the proportion of variance that can be explained by the SNP(s) added to the base model, with adjustment for the number of covariates in the model. A SNP with a *ΔR^2^* of 5% or higher was deemed significant. Effect size (i.e., absolute BMD change) per copy of the variant allele and 95% confidence intervals (CI) were calculated for each significant SNP, with and without adjustment for clinical risk factors. The rate of annualized BMD change between pre- and post-HCT DXA scans was compared and plotted by the genotypes of each significant SNP. To explore potential functionality of the significant SNPs, the SNP and CNV Annotation Database (SCAN) [23] was surveyed to explore whether it is a potential expression quantitative trait loci (eQTL) that has been associated with altered gene expression.

#### Multiple-SNP analysis

We examined the combined effects of multiple variants using backward elimination. SNPs with a p≤0.05 were retained in the final models. Adjusted *R^2^* was calculated for the complete models with both clinical and genetic factors and compared to the base models with only clinical factors, to determine the overall contribution of genetic factors. For SNPs retained in the model, the number of risk alleles was summed for each patient, and the annualized rate of BMD change was compared across patients by number of risk alleles. Although we measured change in BMD between a set interval of approximately 100 days, we presented annualized BMD loss rates for comparisons to the general population. Prior reports of BMD loss post-HCT demonstrated that reversal of BMD loss takes several years after HCT [3]. Multiple-SNP analysis was not performed for spinal BMD loss after allogeneic HCT because only one SNP was retained in the final comprehensive model. All analyses were two-tailed and were performed with SAS 9.2 (SAS Institute, Cary, NC).

## Results

### Individual SNP analysis

Table 1 summarizes the demographic and clinical characteristics of the study population separately by auto- and allo-HCT groups, which were statistically similar to the overall patient population described in our prior report [6]. Unadjusted p-values for associations between individual SNPs and BMD change between pre- and post-HCT are shown in Figure S1. After controlling for clinical risk factors, at least one SNP remained significant for each of the four outcomes, with a total of 16 significant SNPs for all four outcomes. SNP rs2075555 in the *COL1A1* gene remained significant after Bonferroni correction for multiple comparisons (p = 0.037).

**Table 1:** Characteristics of HCT patients with BMD measurement and genomic DNA samples.

Characteristics	Autologous HCT (n = 67)	Allogeneic HCT (n = 54)
Age at HCT in years	57 (25–74)	47 (18–71)
Spine BMD at baseline, median (range), g/cm^2^	1.23 (0.71–1.65)	1.20 (0.86–1.83)
Femur BMD at baseline, median (range), g/cm^2^	1.04 (0.60–1.41)	1.09 (0.71–1.45)
Gender		
Male	42 (63%)	30 (56%)
Female	25 (37%)	24 (44%)
Race		
White	59 (88%)	52 (96%)
Other	8 (12%)	2 (4%)
Diagnosis		
Acute leukemia	3 (4%)	34 (63%)
Lymphoma	37 (55%)	9 (17%)
Myeloma	27 (40%)	0
Other	0	11 (20%)
Disease status at HCT		
CR1/untreated MDS	29 (43%)	38 (70%)
CR2+/PIF/Relapse	38 (57%)	16 (30%)
Graft source		
Bone marrow ± PBSC	6 (9%)	8 (15%)
PBSC	61 (91%)	46 (85%)
Conditioning regimen		
Myeloblative	67 (100%)	19 (35%)
Reduced Intensity	0	32 (59%)
Non-myeloblative	0	3 (6%)
GvHD prophylaxis regimen		
CsMt/FKMt	-	19 (35%)
FK/FKMMF	-	15 (28%)
FKMtMMF		20 (37%)
Donor relation		
Unrelated	-	36 (67%)
Related	-	18 (33%)
HLA match		
Matched	-	45 (83%)
Mismatched	-	9 (17%)
Acute GvHD		
Grade 0–I	-	26 (48%)
Grade II–IV	-	28 (52%)

*Abbreviations:* BMD, bone mineral density; CR, complete remission; Cs, cyclosporine; FK, tacrolimus; GvHD, graft-versus-host disease; HCT, hematopoietic cell transplantation; HLA, human leukocyte antigen; MDS, myelodysplastic syndrome; MMF, mycophenolate mofetil; Mt, methotrexate; PIF, primary induction failure; PBSC, peripheral blood stem cell.

**Figure 1:**
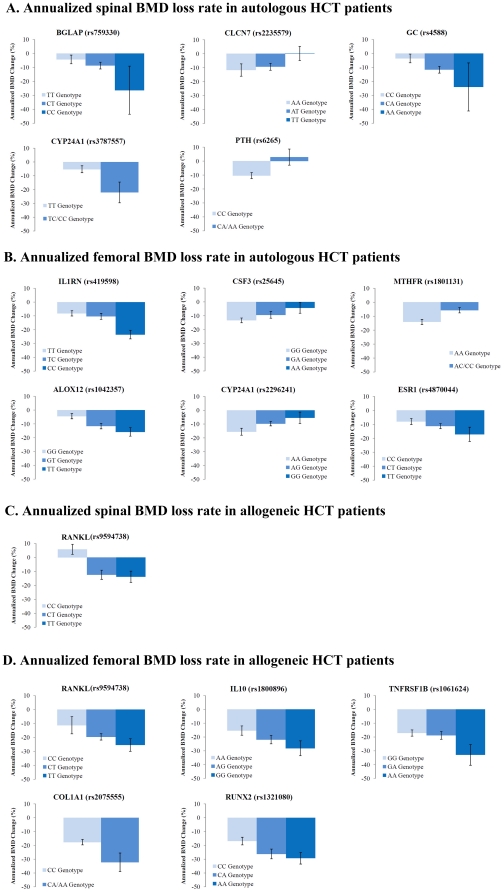
Annualized rate of bone mineral density change after hematopoietic cell transplantation by genotype for SNPs significant in the multivariable models including clinical risk factors. Mean and standard error of the annualized rate of BMD change is plotted by genotype for significant SNPs in the multivariable models including clinical risk factors. If the frequency of the homozygous variant genotype was ≤5%, it was combined with the heterozygous genotype (includes rs3787557 [*CYP24A1*], rs6265 [*PTH*], rs1801131 [*MTHFR*] and rs2075555 [*COL1A1*]). Please refer to Table S2 for a description of functions of these genes and SNPs. *Abbreviation:* BMD, bone mineral density; HCT, hematopoietic cell transplantation; SNP, single nucleotide polymorphism.

Table 2 summarizes the unadjusted and adjusted BMD change associated with each of the 16 SNPs significant in individual SNP tests. Annualized rate of BMD change by genotype are shown in Figure 1. There was a significant dose-response trend between rate of BMD loss and number of risk alleles at the spine and the femur in autologous HCT and at the femur in allogeneic HCT. The functions of these genes, SNPs and published literature are summarized in Table S2. Among these, three SNPs, including rs2075555 in *COL1A1* gene [11], [24], rs9594738 in *RANKL* gene [13] and rs4870044 in *ESR1* gene [13], were significantly associated with bone phenotypes in GWA studies of the general population. Moreover, rs2075555 and rs9594738 were associated with altered gene expression in the SCAN (p<0.0001). Another 9 significant SNPs in our analysis were previously linked to bone phenotypes in candidate gene association studies, including rs4588 in *GC*, rs6256 in *PTH*, rs419598 in *IL1RN*, rs1801131 in *MTHFR*, rs1800896 in *IL10*, and rs1061624 in *TNFRSF1B*, rs1042358 in *ALOX12*, rs759330 in *BGLAP*, and rs2235579 in *CLCN7*. Four of these SNPs (rs759330, rs2235579, rs1800896 and rs1061624) were also associated with altered gene expression in the SCAN. The other 4 SNPs, including rs3787557 and rs2296241 in *CYP24A1*, rs25645 in *CSF3*, and rs1321080 in *RUNX2*, were selected as tagSNPs or SNPs in functional genomic regions, with no known associations with bone phenotypes. Two of these SNPs (rs2296241 and rs25645) were associated with altered gene expression in the SCAN.

**Table 2:** Change in bone mineral density per copy of the variant allele for individual SNPs which were significant in the multivariable models including clinical risk factors^1^.

Gene	SNP	Unadjusted BMD change per copy of variant allele (95% CI), g/cm^2^	Unadjusted P-value	Adjusted BMD change per copy of variant allele (95% CI), g/cm^2^	Adjusted P-value
**Spinal bone mineral density change after autologous hematopoietic cell transplantation**					
*BGLAP*	rs759330	−0.024 (−0.045−0.002)	0.03	−0.026 (−0.047−0.006)	0.01
*CLCN7*	rs2235579	0.016 (−0.001–0.034)	0.07	0.019 (0.002–0.036)	0.03
*GC*	rs4588	−0.031 (−0.051−0.011)	0.003	−0.024 (−0.045−0.003)	0.02
*CYP24A1*	rs3787557	−0.055 (−0.095−0.016)	0.007	−0.049 (−0.087−0.010)	0.01
*PTH*	rs6256	0.036 (0.004–0.067)	0.03	0.032 (0.002–0.063)	0.04
**Femoral bone mineral density change after autologous hematopoietic cell transplantation**					
*IL1RN*	rs419598	−0.014 (−0.026−0.003)	0.01	−0.014 (−0.025−0.004)	0.009
*CSF3*	rs25645	0.011 (0.002–0.021)	0.02	0.011 (0.002–0.020)	0.02
*MTHFR*	rs1801131	0.022 (0.009–0.036)	0.001	0.020 (0.08–0.033)	0.008
*ALOX12*	rs1042357	−0.016 (−0.027−0.005)	0.004	−0.014 (−0.025−0.003)	0.01
*CYP24A1*	rs2296241	0.014 (0.004–0.024)	0.01	0.014 (0.004–0.024)	0.01
*ESR1*	rs4870044	−0.011 (−0.023−0.000)	0.06	−0.015 (−0.025−0.004)	0.008
**Spinal bone mineral density change after allogeneic hematopoietic cell transplantation**					
*RANKL*	rs9594738	−0.029 (−0.052−0.007)	0.01	−0.020 (−0.039−0.001)	0.04
**Femoral bone mineral density change after allogeneic hematopoietic cell transplantation**					
*RANKL*	rs9594738	−0.022 (−0.042−0.003)	0.02	−0.018 (−0.036−0.001)	0.05
*IL10*	rs1800896	−0.016 (−0.035−0.002)	0.08	−0.022 (−0.039−0.005)	0.01
*TNFRSF1B*	rs1061624	−0.023 (−0.041−0.004)	0.02	−0.021 (−0.039−0.003)	0.02
*COL1A1*	rs2075555	−0.052 (−0.080−0.024)	0.0003	−0.050 (−0.077−0.023)	0.0006
*RUNX2*	rs1321080	−0.028 (−0.051−0.006)	0.02	−0.024 (−0.047−0.002)	0.03

*Footnote:*

1 The clinical risk factors included in the multivariable models are: (1) Spinal BMD change after autologous HCT: spinal BMD at baseline and diagnosis (lymphoma vs others); (2) Femoral BMD change after autologous HCT: age at HCT and diagnosis (lymphoma vs others); (3) Spinal BMD change after allogeneic HCT: spinal BMD at baseline, weight at baseline, and steroid dose; (4) Femoral BMD change after allogeneic HCT: femoral BMD at baseline, weight at baseline, and steroid dose. Please refer to Table S2 for description of functions of these genes and SNPs. *Abbreviation:* BMD, bone mineral density; CI, confidence interval; HCT, hematopoietic cell transplantation; SNP, single nucleotide polymorphism.

### Multiple-SNP analysis

The multiple-SNP models are shown in Table 3. For the spinal BMD change after autologous HCT, three SNPs (rs759330 in *BGLAP*, rs4588 in *GC*, and rs3787557 in *CYP24A1*) remained in the final model and explained 16% of the variance in BMD loss, in addition to the 10% determined by the clinical risk factors. Similarly, SNPs retained in the final models explained an additional 35%, 5% and 29% of the variance in femoral BMD loss after autologous HCT, spinal BMD loss after allogeneic HCT, and femoral BMD loss after allogeneic HCT, respectively.

**Table 3:** Multiple SNP models of bone mineral density change after hematopoietic cell transplantation.

Outcome	Base model of clinical factors	Adjusted *R^2^*	SNP (Gene)	Adjusted *R^2^*	*ΔR^2^*
Spinal BMD change after autologous HCT	Spinal BMD at baseline; lymphoma vs others	0.10	rs759330 (*BGALP*); rs4588 (*GC*); rs3787557 (*CYP24A1*)	0.26	0.16
Femoral BMD change after autologous HCT	Age at HCT; lymphoma vs others	0.12	rs419598 (*IL1RN*); rs1801131(*MTHFR*); rs1042357 (*ALOX12*); rs4870044 (*ESR1*)	0.46	0.35
Spinal BMD change after allogeneic HCT	Weight at HCT; spinal BMD at baseline; cortico-steroid dose	0.35	rs9594738 (*RANKL*)	0.40	0.05
Femoral BMD change after allogeneic HCT	Weight at HCT; femoral BMD at baseline; cortico-steroid dose	0.14	rs9594738 (*RANKL*); rs1061624 (*TNFRSF1B*); rs2075555(*COL1A1*); rs1321080 (*RUNX2*)	0.45	0.29

*Footnote: R^2^*, coefficient of determination, provides an estimate of the proportion of BMD change that can be accounted for by all covariates in the model. *ΔR^2^* denotes the increase in model accountability by adding SNP(s) to the base clinical risk factor model. All *R^2^* are adjusted by the number of covariates in the model. A higher *ΔR^2^* suggests an important role of genetics in addition to clinical risk factors for HCT-inducted BMD loss. Please refer to Table S2 for a description of the functions of these genes and SNPs. *Abbreviation:* BMD, bone mineral density; HCT, hematopoietic cell transplantation; SNP, single nucleotide polymorphism.

single nucleotide polymorphism.

**Figure 2:**
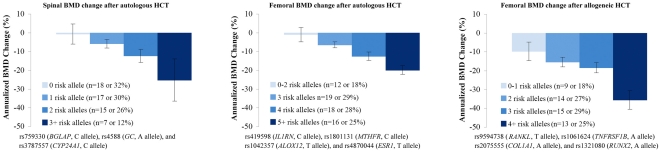
Annualized rate of bone mineral density change after hematopoietic cell transplantation by the number of risk alleles of the SNPs significant in the multiple SNP models. Mean and standard error of annualized rate of BMD change is plotted by the number of risk alleles in significant SNPs from the multiple SNP models as shown in Table 3. The p-values for a trend between the number of risk alleles and BMD change were ≤0.003 for all three outcomes. The proportion of HCT patients carrying the specified number of risk alleles is shown. The multiple SNP analysis was not performed for spinal BMD change after allogeneic HCT since only one SNP was significant in the multiple SNP model. Please refer to Table S2 for a description of these genes and SNPs. *Abbreviation:* BMD, bone mineral density; HCT, hematopoietic cell transplantation; SNP.

When the annualized rate of BMD loss was compared by the number of risk alleles, there was a significant association between higher number of risk alleles and higher BMD loss (Figure 2). In addition, the multiple-SNP models identified patients at the highest risk of BMD loss. Patients carrying the highest number of risk alleles had the most severe BMD loss.

## Discussion

Our analysis identified a number of SNPs in bone metabolism pathways predictive for acute BMD loss following HCT. Because our candidate genes and SNPs have been previously implicated in age-related bone loss, osteoporosis and/or bone phenotypes in the general population, our findings support the hypothesis that HCT-related BMD loss is an acceleration of the normal bone aging process. Moreover, SNPs showed greater effects in HCT patients in our study than those identified in the general population for bone phenotypes. Four SNPs accounted for 35% of variance in femoral BMD change after autologous HCT, whereas the 12 most significant loci in a GWA study accounted for only about 3% of variance in hip and spine BMD in a healthy Icelandic population [13]. The stronger effect may be driven by potent exposures during HCT.

Among the 16 SNPs significant in our analysis, only rs9594738 in *RANKL* gene was related to BMD loss at both the spine and the femur in allogeneic patients. This marginal overlap could be attributed to differences in exposures related to allogeneic versus autologous transplant procedures, as well as to differences in susceptibility to HCT-induced BMD loss at these two anatomic sites. The trabecular interconnection of the lumbar spine is much higher than that of the femur [25]. Thus, the difference in bone microarchitecture may explain the lack of overlap of SNPs associated with BMD and risk of fracture at the two sites in previous studies in the general populations [10] and the observations that femoral bone was more susceptible to HCT-induced bone loss than spinal bone [6], [26].

A limitation of our study comes from the relatively small sample size and the large number of tests performed: 122 SNPs in relation to 4 quantitative outcomes in 121 patients stratified by autologous and allogeneic HCT. The statistical power was thus limited. Instead of using a Bonferroni correction, which would be over conservative, we propose a hierarchy of evidence in our findings based on curated knowledge of the SNPs in the literature.

The first level of evidence includes three SNPs implicated in two GWA studies of bone phenotypes. We found that rs2075555 in the intronic region of *COL1A1* is associated with femoral BMD loss after allogeneic HCT. The gene encodes for type I collagen, a major matrix protein for bone formation and this SNP has been previously associated with femoral neck width in women and shaft width in men [11]. Two other SNPs, rs9594738 near *RANKL* and rs4870044 near *ESR1* from a GWA study of BMD in an Icelandic population [13], and a meta-analysis of GWA studies of BMD^14^, were also significant in our study. *RANKL* plays a central role in coupling the activities of osteoblasts and osteoclasts; and *ESR1* encodes for estrogen receptor alpha, which is key in estrogen-regulated activities.

The second level of evidence includes 9 SNPs, which have been previously studied in relation to various bone phenotypes using a candidate gene approach: rs4588 in *GC*, rs6256 in *PTH*, rs419598 in *IL1RN*, rs1801131 in *MTHFR*, rs1800896 in *IL10*, and rs1061624 in *TNFRSF1B*, and rs1042358 in *ALOX12*, rs759330 in *BGLAP*, and rs2235579 in *CLCN7* (Table S2). The first 5 SNPs are among the most widely studied candidates for osteoporosis and/or other bone phenotypes.

The third level of evidence includes 4 SNPs which have not been previously studied in relation to bone phenotypes: rs3787557 and rs2296241 in *CYP24A1*, rs25645 in *CSF3*, and rs1321080 in *RUNX2*. These SNPs are either tagSNPs or located in potentially functional regions, and these genes were selected for their importance in bone metabolism (Table S2).

As discussed above, we relied on *a priori* knowledge to rank our confidence in our results: those previously implicated in GWA studies were deemed most reliable, followed by those implicated in candidate gene studies, and lastly those without support from the literature. Although this knowledge-based hierarchy of evidence is not statistically stringent, and we cannot exclude the possibility of false positive (or negative) findings in our results, it aligns with our exploratory purpose and provides support that genetic variants contribute to post-HCT bone loss. Moreover, our findings may provide clues for future studies. Thus, in addition to rs2075555 in *COL1A1*, which remained significant in our analysis under the most stringent threshold of significance, we also reported other SNPs that may be interesting. Our study was designed to evaluate only one post-HCT BMD measurement (about +100 days); thus we do not know how the genetic variants described in our study affect long-term BMD post-HCT. We chose day +100 for post-HCT BMD measurement for three reasons. First, BMD loss occurs at the highest rate within the first 3–6 months after HCT [3], thus a true gene-exposure influence would be strongest in the time period closest to the exposure. Second, patients who were osteoporotic at their day +100 DXA scan were treated with a bisphosphonate soon afterwards which may distort the association between genotypes and BMD loss. Third, as an exploratory study, BMD loss at day +100 was an outcome immediately available, while we continue to follow-up patients with the plan to assess genotypes and long-term BMD loss.

It is of interest to investigate the association of donor genotype with BMD loss in allogeneic HCT recipients, considering their hematopoietic cells, and thus osteoclast precursors, are replaced by donors' hematopoietic cells once complete donor chimerism has been achieved. Unfortunately, donor DNA is not available from the allogeneic HCT patients in this study. However, given that the acute BMD loss we observed occurred quickly following transplantation, prior to complete donor chimerism, and that the acute BMD loss in our study was similar between autologous and allogeneic HCT recipients (ie., in the absence and presence of donor genomes), any effect of donor genotype may only be evidenced in chronic BMD loss with longer follow-up. This question warrants further investigation.

In summary, given the continuous bone loss due to normal aging, accelerated post-HCT bone loss may never recover without aggressive therapy, and HCT patients may be at much higher risk of fracture compared to their age-matched peers. Considering the well established relationship between low BMD and fracture risk in the general population, we support the routine post HCT surveillance guidelines developed by McClune et al [17]. Further studies are needed to determine the impact and timing of different prevention and/or intervention strategies.

## Supporting Information

Figure S1 Log-transformed p-values for associations between bone mineral density change and individual SNPs without adjustment for clinical risk factors. Individual SNP (gene) remained significant in the multivariable models including clinical risk factors are labeled in circles. A number of other SNPs significant in univariate analysis but dropped out of multivariate analysis were not labeled. Please refer to Table S2 for description of functions of these genes and SNPs. *Abbreviation:* HCT, hematopoietic cell transplantation; SNP, single nucleotide polymorphism.
(TIF)

Table S1 Selected 122 single nucleotide polymorphisms from 46 candidate genes in bone metabolism pathways. ^1^SNPs that have been previously studied in the large prospective study Genetic Markers for Osteoporosis (GENOMOS) Consortium were labeled as “GENOMOS SNPs”. SNPs that have been identified in genome wide association (GWA) studies were labeled as “GWAS SNP”. TagSNPs selected to represent genetic variations in genes that have not been previous studied and have no common SNPs in functional regions were labeled as “tagSNP”. Functional regions include 3′ and 5′ untranslated region (UTR) and codon. For coding SNPs, amino acid changes are shown.
(DOC)

Table S2 Functions and summary of the published literature for genes and SNPs significant in the individual SNP models including clinical risk factors
(DOC)
