# Supplementary figures and images for: Common Genetic Variants Are Associated with Accelerated Bone Mineral Density Loss after Hematopoietic Cell Transplantation
Source: PLoS One. 2011 Oct 14;6(10):e25940. doi: 10.1371/journal.pone.0025940 (PMC3195081; doi:10.1371/journal.pone.0025940)

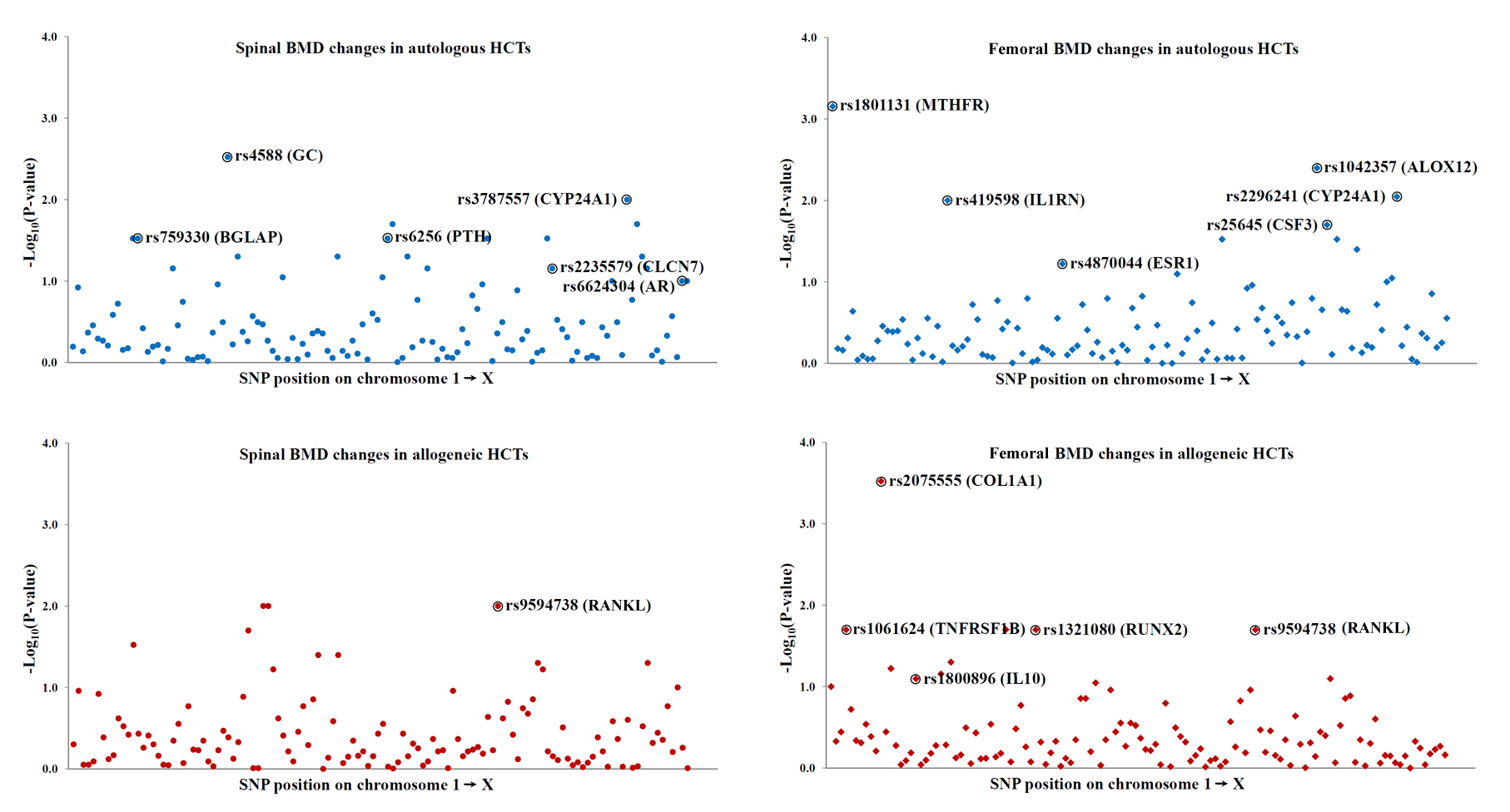

Supplement: Figure S1 — Log-transformed p-values for associations between bone mineral density change and individual SNPs without adjustment for clinical risk factors. Individual SNP (gene) remained significant in the multivariable models including clinical risk factors are labeled in circles. A number of other SNPs significant in univariate analysis but dropped out of multivariate analysis were not labeled. Please refer to Table S2 for description of functions of these genes and SNPs. Abbreviation: HCT, hematopoietic cell transplantation; SNP, single nucleotide polymorphism. (TIF) [file pone.0025940.s001.tif]
