# Supplementary material for: Common Genetic Variants Are Associated with Accelerated Bone Mineral Density Loss after Hematopoietic Cell Transplantation
Source: PLoS One. 2011 Oct 14;6(10):e25940. doi: 10.1371/journal.pone.0025940 (PMC3195081; doi:10.1371/journal.pone.0025940)
Supplement: Table S1 — Selected 122 single nucleotide polymorphisms from 46 candidate genes in bone metabolism pathways. 1SNPs that have been previously studied in the large prospective study Genetic Markers for Osteoporosis (GENOMOS) Consortium were labeled as “GENOMOS SNPs”. SNPs that have been identified in genome wide association (GWA) studies were labeled as “GWAS SNP”. TagSNPs selected to represent genetic variations in genes that have not been previous studied and have no common SNPs in functional regions were labeled as “tagSNP”. Functional regions include 3′ and 5′ untranslated region (UTR) and codon. For coding SNPs, amino acid changes are shown. (DOC) [file pone.0025940.s002.doc]

Table S1. Selected 122 single nucleotide polymorphisms from 46 candidate genes in bone metabolism pathways

| **Gene symbol** | **Full name** | **Locus** | **SNP** | **Function^1^** |
| --- | --- | --- | --- | --- |
| **Pathway 1: RANKL-RANK-OPG central signaling axis and regulating cytokines and receptors** | | | | |
| *RANKL* | Receptor activator of nuclear factor-kappa B ligand (TNFSF11) | 13q14 | rs1054016 | 3' UTR |
|  |  |  | rs9525641 | Near gene 5' end |
|  |  |  | rs3742257 | Intronic |
|  |  |  | rs9594738 | GWAS SNP |
|  |  |  | rs9594759 | GWAS SNP |
|  |  |  | rs9594782 | Intronic |
| *RANK* | Receptor activator of nuclear factor-kappa B (TNFRSF11A) | 18q22.1 | rs1805034 | Val192Ala |
| *OPG* | Osteoprotegrin (TNFRSF11B) | 8q24.1 | rs3102735 | Near gene 5' end |
|  |  |  | rs4355801 | GWAS SNP |
|  |  |  | rs6993813 | GWAS SNP |
|  |  |  | rs6469804 | GWAS SNP |
| *IL1A* | Interleukin 1 alpha | 2q14 | rs17561 | Ser114Ala |
| *IL1B* | Interleukin 1 beta | 2q14 | rs1071676 | 3' UTR |
|  |  |  | rs16944 | -1060T>C |
| *IL1R1* | Interleukin 1 receptor type 1 | 2q12 | rs2228139 | Gly124Ala |
|  |  |  | rs2110726 | 3' UTR |
| *IL1RN* | Interleukin 1 receptor antagonist | 2q14.2 | rs419598 | Ala39Ala |
|  |  |  | rs315952 | Ser112Ser |
| *IL2* | Interleukin 2 | 4q26-q27 | rs2069762 | -384G>T |
|  |  |  | rs2069763 | Leu38Leu |
| *IL4* | Interleukin 4 | 5q31.1 | rs2243250 | Near gene 5' end |
| *IL6* | Interleukin 6 | 7q21 | rs1800797 | -660A>G |
| *IL10* | Interleukin 10 | 1q31-32 | rs1800871 | -853C>T |
|  |  |  | rs1800896 | -1116A>G |
| *TNF* | Tumor necrosis factor | 6p21.3 | rs1799964 | Near gene 5' end |
|  |  |  | rs1800629 | Near gene 5' end |
| *TNFRSF1B* | Tumor necrosis factor receptor 2 | 1p36.3-36.2 | rs1061622 | Arg196Met |
|  |  |  | rs1061624 | 3' UTR |
|  |  |  | rs3397 | 3' UTR |
|  |  |  | rs1061628 | 3' UTR |
|  |  |  | rs1061631 | 3' UTR |
| *PTGS2* | Prostaglandin endo-peroxide synthase 2 (COX2) | 1p25.2-25.3 | rs2745557 | Intronic |
|  |  |  | rs5277 | Val102Val |
| *CSF2* | Colony stimulating factor 2 (GM-CSF) | 5q31.1 | rs25882 | Thr117Ile |
| *CSF3* | Colony stimulating factor 3 (G-CSF) | 17q11.2-q12 | rs25645 | Leu185Leu |
|  |  |  | rs1042658 | 3' UTR |
|  |  |  | rs2827 | 3' UTR |
| *CSF2RB* | Colony stimulating factor 2 receptor beta | 22q13.1 | rs1534881 | tagSNP |
|  |  |  | rs11705394 | tagSNP |
|  |  |  | rs909486 | tagSNP |
|  |  |  | rs2075941 | tagSNP |
| *CSF3R* | Colony stimulating factor 3 receptor | 1p35-p34.3 | rs3917980 | Arg418Arg |
|  |  |  | rs3917924 | tagSNP |
|  |  |  | rs3917979 | tagSNP |
| **Pathway 2: Bone matrix proteins and regulating factors** | | | | |
| *COL1A1* | Collagen type 1, alpha 1 | 17q21.33 | rs1800012 | GENOMOS SNP |
|  |  |  | rs2075555 | GWAS SNP |
| *BGLAP* | Osteocalcin | 1q25-q31 | rs1800247 | Near gene 5' end |
|  |  |  | rs759330 | Near gene 3' end |
| *BMP2* | Bone morphogenetic protein 2 | 20p10 | rs235768 | Ser190Arg |
|  |  |  | rs3178250 | 3' UTR |
|  |  |  | rs170986 | 3' UTR |
| *CASR* | Calcium-sensing receptor | 3q13 | rs1801725 | Ser986Ala |
|  |  |  | rs1042636 | Gly900Arg |
| *CALCA* | Calcitonin | 11p15.2-15.1 | rs5242 | Intronic |
| *CALCR* | Calcitonin receptor | 7q21.3 | rs1801197 | Pro447Leu |
|  |  |  | rs1042138 | 3' UTR |
|  |  |  | rs2301680 | 5' UTR |
| *TGFB1* | Transforming growth factor beta 1 | 19q13.1 | rs1800469 | GENOMOS SNP |
| *IGF1* | Insulin-like growth factor 1 | 12q22-23 | rs5742632 | tagSNP |
|  |  |  | rs1019731 | tagSNP |
|  |  |  | rs6214 | tagSNP |
|  |  |  | rs7136446 | tagSNP |
|  |  |  | rs1520220 | tagSNP |
| *LRP5* | Low density lipoprotein receptor-related protein 5 | 11q13.4 | rs3736228 | GENOMOS SNP |
|  |  |  | rs556442 | Val1119Val |
|  |  |  | rs2306862 | Asn740Asn |
|  |  |  | rs4988300 | GWAS SNP |
| *RUNX2* | Runt-related transcription factor 2 | 6p21 | rs13201287 | tagSNP |
|  |  |  | rs1321080 | tagSNP |
|  |  |  | rs6458446 | tagSNP |
|  |  |  | rs2819863 | tagSNP |
| *APOE* | Apolipoprotein E | 19q13.2 | rs7412 | Cys176Arg |
|  |  |  | rs429358 | Arg130Cys |
| *MTHFR* | Methylenetetrahydrofolate reductase | 1p36.3 | rs1801131 | Ala429Glu |
|  |  |  | rs1801133 | GWAS SNP |
| *ALOX12* | Arachidonate 12-lipooygenase | 17p13.1 | rs1042357 | Thr364Thr |
|  |  |  | rs312462 | Leu634Leu |
| *CLCN7* | Chloride channel 7 | 16p13 | rs12926669 | Ile415Ile |
|  |  |  | rs2235579 | Ala390Ala |
| *SERPINE1* | Serpin peptidase inhibitor clade E member 1 | 7q21.3-q22 | rs6092 | Thr15Ala |
| **Pathway 3: Vitamin D receptor and metabolism enzymes** | | | | |
| *VDR* | Vitamin D receptor | 12q13.11 | rs11568820 | GENOMOS SNP |
|  |  |  | rs4516035 | Near gene 5' end |
|  |  |  | rs2228570 | GENOMOS SNP |
|  |  |  | rs7975232 | GENOMOS SNP |
|  |  |  | rs731236 | GENOMOS SNP |
|  |  |  | rs2189480 | GWAS SNP |
| *GC* | Vitamin D binding protein | 4q12-q13 | rs4588 | Lys436Thr |
|  |  |  | rs7041 | Glu432Asp |
| *CYP24A1* | 24-hydroxylase | 20q13 | rs2296241 | tagSNP |
|  |  |  | rs3787557 | tagSNP |
|  |  |  | rs6013905 | tagSNP |
|  |  |  | rs4809960 | tagSNP |
|  |  |  | rs912505 | tagSNP |
| *CYP27A1* | 25-hydroxylase | 20q33 | rs4674344 | tagSNP |
|  |  |  | rs17470271 | tagSNP |
| *CYP27B1* | 1alpha-hydroxylase | 12q13.1-q13.3 | rs4646536 | Intronic |
| **Pathway 4: Steroid hormones and receptors** | | | | |
| *ESR1* | Estrogen receptor 1 | 6q25.1 | rs9340799 | GENOMOS SNP |
|  |  |  | rs2234693 | GENOMOS SNP |
|  |  |  | rs1884052 | GWAS SNP |
|  |  |  | rs9479055 | GWAS SNP |
|  |  |  | rs4870044 | GWAS SNP |
|  |  |  | rs1038304 | GWAS SNP |
|  |  |  | rs6929137 | GWAS SNP |
|  |  |  | rs1999805 | GWAS SNP |
| *CYP17A1* | Cytochrome P450, family 17 | 10q24.3 | rs743572 | 5' UTR |
| *CYP19A1* | Aromatase | 15q21.1 | rs700518 | Val80Val |
|  |  |  | rs4646 | 3' UTR |
|  |  |  | rs10046 | 3' UTR |
|  |  |  | rs10519297 | GWAS SNP |
|  |  |  | rs2008691 | GWAS SNP |
| *COMT* | Catechol-O-methyltransferase | 22q11.21 | rs4680 | Met158Val |
|  |  |  | rs4818 | Leu136Leu |
| *PTH* | Parathyroid hormone | 11p15.3-15.1 | rs192802 | tagSNP |
|  |  |  | rs751610 | tagSNP |
|  |  |  | rs6254 | tagSNP |
|  |  |  | rs6256 | Arg81Arg |
| *PTHR1* | Parathyroid hormone receptor 1 | 3p22-p21.1 | rs724449 | tagSNP |
|  |  |  | rs724450 | tagSNP |
|  |  |  | rs6442037 | tagSNP |
|  |  |  | rs1138518 | Asn463Asn |
| *NR3C1* | Glucocorticoid receptor | 5q31.3 | rs6196 | Asn766Asn |
|  |  |  | rs6191 | 3' UTR |

*Footnote:* ^1^ SNPs that have been previously studied in the large prospective study Genetic Markers for Osteoporosis (GENOMOS) Consortium were labeled as “GENOMOS SNPs”. SNPs that have been identified in genome wide association (GWA) studies were labeled as “GWAS SNP”. TagSNPs selected to represent genetic variations in genes that have not been previous studied and have no common SNPs in functional regions were labeled as “tagSNP”. Functional regions include 3’ and 5’ untranslated region (UTR) and codon. For coding SNPs, amino acid changes are shown.
