# Supplementary material for: Common Genetic Variants Are Associated with Accelerated Bone Mineral Density Loss after Hematopoietic Cell Transplantation
Source: PLoS One. 2011 Oct 14;6(10):e25940. doi: 10.1371/journal.pone.0025940 (PMC3195081; doi:10.1371/journal.pone.0025940)
Supplement: Table S2 — Functions and summary of the published literature for genes and SNPs significant in the individual SNP models including clinical risk factors (DOC) [file pone.0025940.s003.doc]

Table S2. Functions and summary of the published literature for genes and SNPs significant in the individual SNP models including clinical risk factors

| Gene symbol | Protein function in bone metabolism | SNP | SNP function | Previous study associations (reference) |
| --- | --- | --- | --- | --- |
| *COL1A1* | Type I collagen fiber component, a major bone protein | rs2075555 | Intronic; eQTL in the SCAN database (p<0.0001); GWAS SNP | Bone phenotypes and breast cancer risk in genome-wide scans (1, 2). |
| *RANKL* | Central signaling axis coupling activities of osteoblasts and osteoclasts. | rs9594738 | Close to *TNFSF11* genetic region; eQTL in the SCAN database (p<0.0001); GWAS SNP | Spinal and femoral BMD in a genome-wide scan (3). |
| *ESR1* | Estrogen receptor, an estrogen activated transcription factor | rs4870044 | Close to *ESR1* genetic region; GWAS SNP | Spinal and femoral BMD in a genome-wide scan (4). |
| *GC* | Vitamin D binding protein and transporter in circulation | rs4588 | Non-synonymous coding SNP, Lys436Thr. | Gc-globulin levels and serum 25OHD levels (4, 5), BMD and risk of osteoporosis (6). |
| *PTH* | Parathyroid hormone, essential for calcium homeostasis | rs6256 | Synonymous coding SNP, Arg83Arg | BMD, risk of fracture and hyperparathyroidism (7, 8). |
| *IL1RN* | Interleukin 1 antagonist, regulating immune and inflammatory responses | rs419598 | Synonymous coding SNP, Ala39Ala; locates within an extended IL1A-IL1B-IL1RN high LD region | Risk of osteoarthritis, graft-versus-host-disease and a variety of inflammatory diseases (9-11). |
| *MTHFR* | Folate reductase, folate metabolism | rs1801131 | Non-synonymous coding SNP, Glu429Ala; in high LD with another commonly studied SNP rs1801133 | BMD, risk of osteoporosis, fracture and a variety of diseases and treatment outcomes (12-14). |
| *IL10* | Cytokine, immunoregulation and inflammation | rs1800896 | 5' near *IL10* gene; eQTL in the SCAN database (p<0.0001) | Lower expression of IL10, risk of rheumatoid arthritis, response to anti-TNF treatment, and graft-versus-host-disease (15-17). |
| *TNFRSF1B* | Tumor necrosis factor receptor, antagonist for TNF-α | rs1061624 | 3' UTR; eQTL in the SCAN database (p<0.0001) | Bone structure, BMD, risk of bone loss and fracture (18, 19). |
| *ALOX12* | Lipoxygenase; gene defects causes osteoporosis | rs1042357 | Synonymous coding SNP, Thr364Thr | BMD and risk of colorectal cancer (20, 21). |
| *BGLAP* | Osteocalcin, bone mineralization and calcium homeostasis | rs759330 | 3' near *BGLAP* gene; eQTL in the SCAN database (p=0.0001). | Serum osteocalcin levels (22). |
| *CLCN7* | Chloride channel 7; gene defects cause osteopetrosis | rs2235579 | Synonymous coding SNP, Ala366Ala; eQTL in the SCAN database (p<0.0001) | BMD and bone resorption markers (23). |
| *CYP24A1* | 24-hydroxylase for vitamin D degradation | rs3787557 | Intronic; tagSNP |  |
| *CYP24A1* | 24-hydroxylase for vitamin D degradation | rs2296241 | Synonymous coding SNP, Ala950Ala; eQTL in the SCAN database (p<0.0001) |  |
| *CSF3* | Cytokine regulating granulocytes | rs25645 | Synonymous coding SNP, Leu185Leu; eQTL in the SCAN database (p<0.0001) |  |
| *RUNX2* | Transcription factor for osteoblast differentiation and skeletal morphogenesis | rs1321080 | Intronic; tagSNP |  |

References:

1. Kiel DP, Demissie S, Dupuis J, Lunetta KL, Murabito JM, Karasik D. Genome-wide association with bone mass and geometry in the Framingham Heart Study. BMC Med Genet 2007;8 Suppl 1:S14.

2. Murabito JM, Rosenberg CL, Finger D, et al. A genome-wide association study of breast and prostate cancer in the NHLBI's Framingham Heart Study. BMC Med Genet 2007;8 Suppl 1:S6.

3. Styrkarsdottir U, Halldorsson BV, Gretarsdottir S, et al. New sequence variants associated with bone mineral density. Nat Genet 2009;41:15-17.

4. Engelman CD, Fingerlin TE, Langefeld CD, et al. Genetic and environmental determinants of 25-hydroxyvitamin D and 1,25-dihydroxyvitamin D levels in Hispanic and African Americans. J Clin Endocrinol Metab 2008; 93:3381-8.

5. McGrath JJ, Saha S, Burne TH, Eyles DW. A systematic review of the association between common single nucleotide polymorphisms and 25-hydroxyvitamin D concentrations. J Steroid Biochem Mol Biol 2010;121(1-2):471-477.

6. Fang Y, van Meurs JB, Arp P, et al. Vitamin D binding protein genotype and osteoporosis. Calcif Tissue Int 2009;85(2):85-93.

7. Tenne M, McGuigan F, Jansson L, et al. Genetic variation in the PTH pathway and bone phenotypes in elderly women: evaluation of PTH, PTHLH, PTHR1 and PTHR2 genes. Bone 2008;42:719-727.

8. Gohda T, Shou I, Fukui M, et al. Parathyroid hormone gene polymorphism and secondary hyperparathyroidism in hemodialysis patients. Am J Kidney Dis 2002;39:1255-1260.

9. Smith AJ, Keen LJ, Billingham MJ, et al. Extended haplotypes and linkage disequilibrium in the IL1R1-IL1A-IL1B-IL1RN gene cluster: association with knee osteoarthritis. Genes Immun 2004;5:451-460.

10. Moxley G, Han J, Stern AG, Riley BP. Potential influence of IL1B haplotype and IL1A-IL1B-IL1RN extended haplotype on hand osteoarthritis risk. Osteoarthritis Cartilage 2007;15:1106-1112.

11. Sivula J, Turpeinen H, Volin L, Partanen J. Association of IL-10 and IL-10Rbeta gene polymorphisms with graft-versus-host disease after haematopoietic stem cell transplantation from an HLA-identical sibling donor. BMC Immunol. 2009;10:24.

12. Shiraki M, Urano T, Kuroda T, et al. The synergistic effect of bone mineral density and methylenetetrahydrofolate reductase (MTHFR) polymorphism (C677T) on fractures. J Bone Miner Metab 2008;26:595-602.

13. Riancho JA, Valero C, Zarrabeitia MT. MTHFR polymorphism and bone mineral density: meta-analysis of published studies. Calcif Tissue Int 2006;79:289-293.

14. Schwahn B, Rozen R. Polymorphisms in the methylenetetrahydrofolate reductase gene: clinical consequences. Am J Pharmacogenomics 2001;1:189-201.

15. Nemec P, Goldbergova MP, Gatterova J, Vasku A, Soucek M. Association of polymorphisms in interleukin-10 gene promoter with autoantibody production in patients with rheumatoid arthritis. Ann N Y Acad Sci 2009;1173:501-508.

16. Liu C, Batliwalla F, Li W, et al. Genome-wide association scan identifies candidate polymorphisms associated with differential response to anti-TNF treatment in rheumatoid arthritis. Mol Med 2008;14:575-581.

17. Bertinetto FE, Dall'Omo AM, Mazzola GA, et al. Role of non-HLA genetic polymorphisms in graft-versus-host disease after haematopoietic stem cell transplantation. Int J Immunogenet. 2006;33:375-384.

18. Mullin BH, Prince RL, Dick IM, et al. Bone structural effects of variation in the TNFRSF1B gene encoding the tumor necrosis factor receptor 2. Osteoporos Int 2008;19:961-968.

19. Tasker PN, Albagha OM, Masson CB, Reid DM, Ralston SH. Association between TNFRSF1B polymorphisms and bone mineral density, bone loss and fracture. Osteoporos Int 2004;15:903-908.

20. Mullin BH, Spector TD, Curtis CC, et al. Polymorphisms in ALOX12, but not ALOX15, are significantly associated with BMD in postmenopausal women. Calcif Tissue Int 2007;81:10-17.

21. Goodman JE, Bowman ED, Chanock SJ, Alberg AJ, Harris CC. Arachidonate lipoxygenase (ALOX) and cyclooxygenase (COX) polymorphisms and colon cancer risk. Carcinogenesis 2004;25:2467-2472.

22. Hsu Y, Karasik D, Fox C, et al. Serum Osteocalcin Concentrations, Variants in the Osteocalcin Gene, and their Relationship to Diabetes, Adiponectin Concentration and Visceral Adipose Tissue Volume in Adult Men and Women: The Framingham Study. ASBMR Meeting on New Frontiers in Skeletal Research: Bone, Fat and Brain Connections. 2009.

23. Kornak U, Ostertag A, Branger S, Benichou O, de Vernejoul MC. Polymorphisms in the CLCN7 gene modulate bone density in postmenopausal women and in patients with autosomal dominant osteopetrosis type II. J Clin Endocrinol Metab 2006;91:995-1000.
